# The Number and Habitat Use of Mesopredators Based on the Camera Trapping and Location of Burrows in Hungary

**DOI:** 10.3390/life16020187

**Published:** 2026-01-23

**Authors:** Zoltán Horváth, András Vajkai, Mihály Márton

**Affiliations:** Department of Wildlife Biology and Management, Institute for Wildlife Management and Nature Conservation, Hungarian University of Agriculture and Life Sciences, Páter Károly Str. 1, H-2100 Gödöllő, Hungary; vajkai.andras@phd.uni-mate.hu (A.V.); marton.mihaly@uni-mate.hu (M.M.)

**Keywords:** European badger, red fox, golden jackal, burrow density, habitat preference, litter size, individual density, minimum population

## Abstract

The increasing population of mesopredators in Central Europe necessitates precise monitoring for effective game management. This study aimed to estimate the minimum population and reproduction of the European badger (*Meles meles*), red fox (*Vulpes vulpes*), and golden jackal (*Canis aureus*) in two hunting grounds in southwestern Hungary (Drávaszentes and Darány). Methods included a total burrow count conducted in early 2025, followed by the deployment of wildlife cameras at inhabited setts to record adults and cubs. Results indicated an inhabited burrow density of 1.05/100 ha for badgers and 0.38/100 ha for foxes in Drávaszentes, with average litter sizes of 1.13 and 2.33 cubs, respectively. In Darány, badger density was 1.43/100 ha, while jackals were present at 0.2/100 ha. Additionally, habitat composition preference was analysed using QGIS by comparing Corine Land Cover categories within 400 m buffers around burrows against random points. Habitat analysis suggested local preferences for non-irrigated arable land and mixed forests. These findings provide essential baseline data on predator population dynamics to support conscious management decisions.

## 1. Introduction

In habitats where apex predators have been suppressed, mesopredators are receiving increasing attention. In such cases, food chains are shortened and mesopredators often move up to higher levels of the food chain [1,2]. Therefore, the study of mesopredators becomes important for understanding ecological processes [3]. According to the mesopredator release theory, as the population of medium-sized predators increases, so does the predation pressure on prey species. This top-down effect can also cause changes in biodiversity [1]. From the perspective of certain economic sectors (nature conservation, game management), mesopredators also have an economic impact, which manifests itself in several ways. One example is the direct destruction of individuals of species under nature conservation protection as well as small game species. In addition, predator control can also entail significant costs (trapping, firearms, time).

In a significant part of Central Europe, including Hungary, the red fox (*Vulpes vulpes* Linnaeus, 1758), Eurasian badger (*Meles meles* Linnaeus, 1758), and golden jackal (*Canis aureus* Linnaeus, 1758) are considered widespread species of mesopredators. The red fox was the predator with the largest number of individuals in Hungary in the 2000s and 2010s [4]. In addition, the European badger was added to the list of huntable species in 2001 due to its nationwide distribution [5,6]. In the 1980s, Hungary was one of the first countries where the golden jackal reappeared in Europe [7,8]. As no breeding individuals of jackals were recorded between 1940 and 1990, the species was declared extinct in 1989 [9]. In the early 1990s, jackals began to return, and their population grew so rapidly that it became a huntable species in 1994, as a species that could be hunted throughout the year until 1999 and again from 2012 onwards. In between this period, the hunting season was from 1st of July to the end of February [10,11]. In addition to the above-mentioned effects of mesopredators (predation, competition), foxes and badgers are known to spread diseases that pose a risk to animal and human health [12]. Overall, these factors make it important to manage predators in a conscious manner based on measured data. For this reason, understanding the population dynamics and habitat selection of predators is necessary [13,14]. Foxes and badgers use burrows. Therefore, it is useful to know the location of these underground shelters. The results of population estimations based on burrows can be biased by several behavioral factors. For example, badgers can live either alone or in clans [15]. On the other hand, foxes may use several burrows during a given period, which can lead to an overestimation of population size [16]. It is also possible that foxes and badgers share the same burrows or that certain entrances are only used seasonally [17]. The golden jackal can use burrows as well, but in most cases, it has shelter on the ground surface [18]. In this regard, proportional regional differences are noted for different geoecological habitats of these species of mesopredators [19,20]. Due to these factors, it is important to observe the burrows found and integrate the data collected into predator management.

### Hypotheses

We conducted our studies in two differently managed (nature conservation, wildlife management) hunting areas in southwestern Hungary. At a nature conservation area, the main goal is the biodiversity conservation and the maintenance of natural ecological processes, and the hunting is strictly regulated. Any intervention must be minimal and carried out with a lot of precautions. In an area that focuses on wildlife management, the main goal is to obtain sustainable use, and hunting is the core management tool to achieve it.

Based on the above, our study has two main objectives: (1) estimating the minimum number of foxes, badgers and jackals per burrow using wildlife cameras; (2) characterizing the habitat composition in the surrounding areas of the inhabited burrows. Taking into account the aims, our hypotheses were as follows:

1. Most of the inhabited burrows are used by red foxes.
2. The observed litter size of the studied predator species is approximately the same as that reported in the previously published national (Hungary) data.
3. Habitat composition significantly influences burrow use, differing between burrows inhabited with cubs, inhabited burrows without cubs, and random points.

## 2. Materials and Methods

### 2.1. The Study Areas

**Figure 1:**
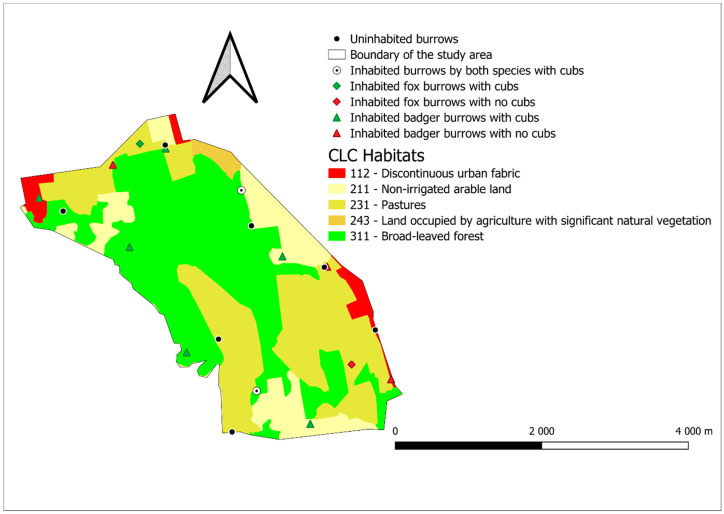
The location of burrows found in the Drávaszentes study area.

The study area in Drávaszentes is located in the southern part of Somogy County, between the towns of Barcs, Komlósd and Péterhida (coordinates: 45.997562561, 17.396221579). The area is a special game management unit managed by the Danube-Drava National Park Directorate. It covers an area of 1138 hectares, of which the hunting area is 1042 hectares. The study area is in the Middle Drava Valley micro-region, on a high floodplain, and lying in the former riverbed of Drava; thus, its located 10–20 m lower than the neighboring areas, and only the eastern side of the area is hilly. The area is mostly plain with only a few meters of elevation difference (lowest: 94 m above sea level, highest: 130 m above sea level). The area has a moderately humid climate, with an average annual temperature of 10.0–10.2 °C and the average annual precipitation is around 780–800 mm. Two small watercourse runs in the study are Györgyös stream and the Komlósdi-Rinya, and they also converge in the area, but both dry up in the drier years. The groundwater is high; it can be found everywhere at around 2–4 m [21]. We calculated the habitats of the area with QGIS (3.30.1) from the Corine Land Cover layer. The habitat with the biggest area is the Broad-leaved forest (CLC 311) covering 46% of the area. Pastures (CLC 231) are also very prominent in the area (34.2%). These are followed by non-irrigated arable lands (CLC 211) with 14,9% coverage, discontinuous urban fabric (CLC 112) with 3.6% and land occupied by agriculture with significant natural vegetation (CLC 243) with 1.3% (Figure 1).

**Figure 2:**
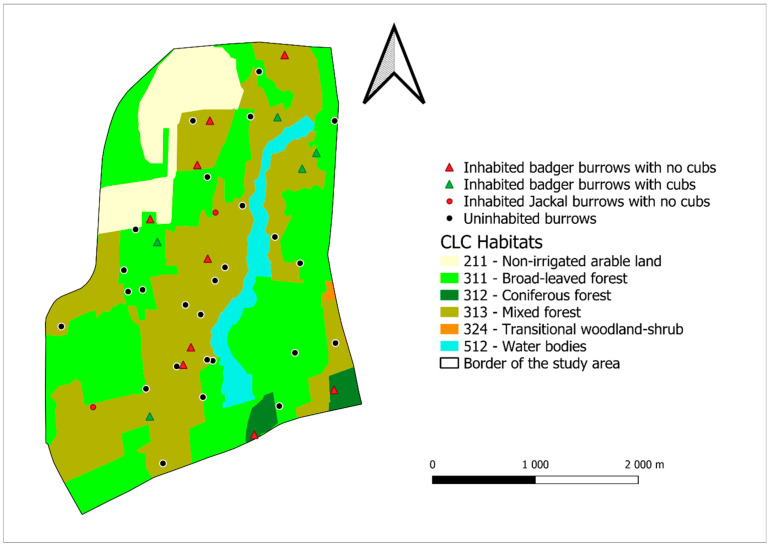
The location of burrows found in the Darány study area.

The study area in Darány is also located in the southern part of Somogy County, between the towns of Barcs and Darány (coordinates: 45.986786021, 17.533621622). It covers an area of 980.8 ha. The study area is in the Eastern-Inner Somogy micro-region. The area is flat, with the lowest point at 125 m above sea level and the highest point at 145 m above sea level. The climate of the area is moderately humid, with the average annual temperature between 10.0 and 10.3 °C. The annual precipitation exceeds 800 mm. Rigóc stream runs through the area, but it dries out in the drier years. The area also has 9 fishponds, only 5 of which contain water, and in the dry period all of them dries out except 2. The remaining ones are reedy-bushy areas. The groundwater can be found between 2 and 4 m [21]. With the same method as the previous study area, we calculated the following habitat covering. The habitat with the biggest coverage is the broad-leaved forest (CLC 311), with 42.4%, followed by the mixed forest (CLC 313) with 40.6%. The remaining area was covered by non-irrigated arable land (CLC 211) with 9.8%, water bodies (CLC 512) with 5.1%, Coniferous forests (CLC 312) with 1.9% and transitional woodland shrubs (CLC 324) with 0.2% (Figure 2).

### 2.2. Data Collection

#### 2.2.1. Field Survey

**Figure A1:**
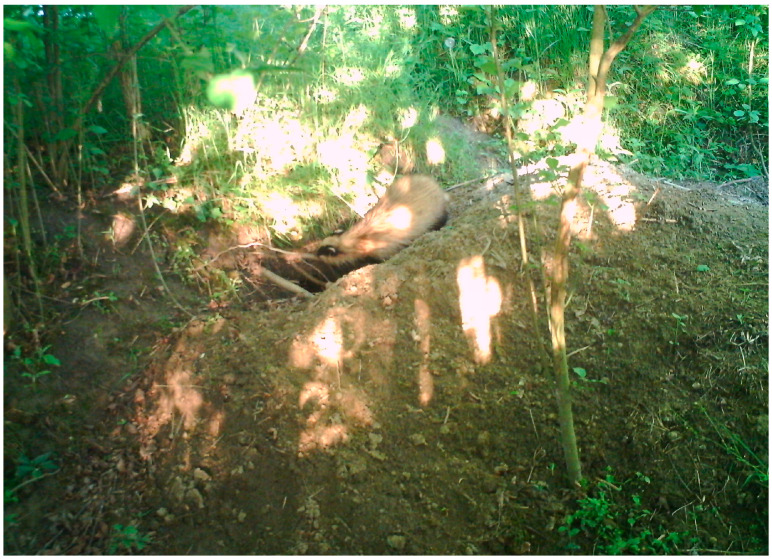
Badger burrow.

**Figure A2:**
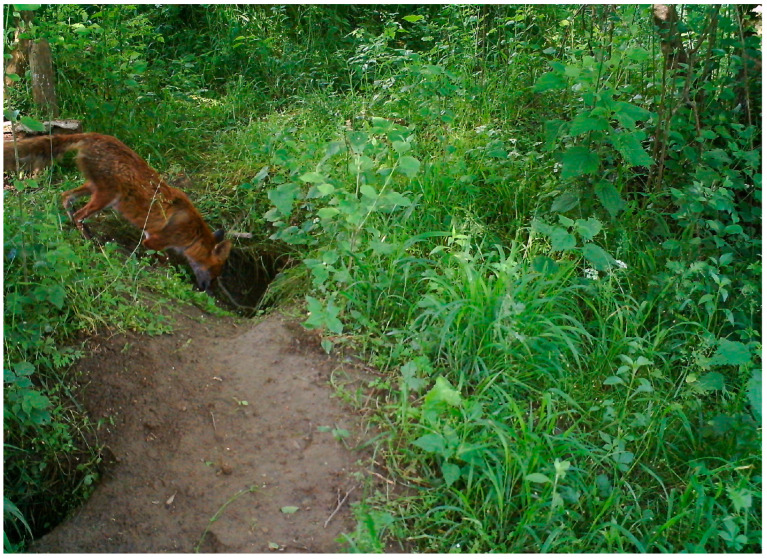
Red fox burrow.

**Figure A3:**
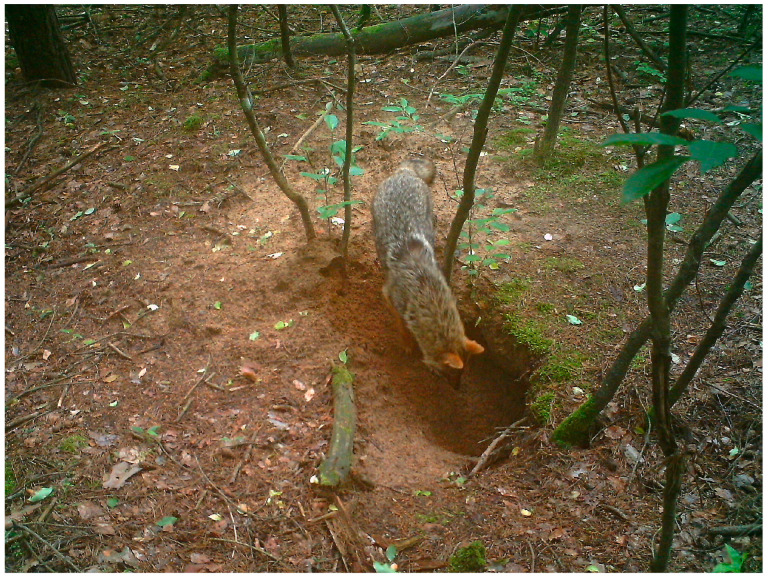
Golden jackal burrow.

To locate the burrow systems, a total count survey was carried out on both study sites to collect the most reliable data possible. The surveys were carried out in 2025 from February to April. Early spring offers optimal visibility of the burrows, because the vegetation has not yet grown dense enough to obscure them. Throughout the fieldwork, maps and a Garmin GPSmap 62s GPS device (Olathe, KS, USA) were used to document every burrow we found. In addition, the coordinate system used was the Unified National Projection System (EOV). The definition of a burrow includes a cavity deeper than one meter, with dimensions appropriate to the body size of the studied species [22]. The entrance generally measured between 15- and 60 cm in width providing a suitable shelter for the badger and red fox [23] (Figure A1, Figure A2 and Figure A3). Where the nearest entrances were more than 50 m apart, we treated them as separate burrow [22]. A field log was maintained to hold the coordinates of each burrow recorded via GPS. This log also contained the serial number, occupancy status and which species were using the given burrow. Species using burrows were identified by using tracks, footprints, scent marks, droppings and latrines [22]. We considered a burrow to be occupied if the entrances appeared to be clean and showed signs of recent activity. As for the badger, there were characteristic indicators that helped to distinguish their burrows from those of other species [24,25,26,27,28].

#### 2.2.2. Survey of Minimum Population and Litter Size Using Wildlife Cameras

Where possible, motion-activated wildlife cameras were installed at burrows identified as inhabited to record the minimum number of adults and juveniles inhabiting them. Camera trapping can provide valuable information on species that are nocturnal or elusive and are difficult to observe [29]. Based on the images captured by wildlife cameras, we determined the largest number of cubs and adults seen at once for each burrow. During the study, we used 6 cameras with image transmission capability and 15 cameras without image transmission capability. The cameras were set to photo mode with a delay of 3 s, and the burst mode was turned off. The quality of the pictures was set to 5 MP and the Passive Infrared Motion sensor (PIR) set to normal. The cameras were put on a nearby tree with a ladder in about 2.5 m height and about 5 m from the active entrance. They were secured to the tree with Python (QGIS Desktop 3.30.1) cables. The burrows at the Drávaszentes study area were surveyed between 18 April 2025 and 6 May 2025 (16 days); at the Darány study area, they were surveyed from 20 May 2025 to 8 June 2025 (19 days). There were also multi-entrance burrow systems, where we installed multiple cameras at different entrances. The models of cameras include Bushnell (Nature View), Reconyx (HC500), Uovison (Easy Mail, Select 30, 565), and Minox (DTC 400, DTC 650).

In the case of large burrows with several entrances, it was not always feasible to position a camera at the specific entrance used by the predators. This presumably meant that it was not always possible to accurately determine the number of adults and juveniles. In open grassland habitats, the risk of illegal appropriation by unauthorized persons was higher, which limited the use of cameras in these areas. In such cases, we determined the minimum number of individuals based on the tracks observed. In total, 31 burrows were monitored with cameras, 15 at the Drávaszentes study area, and 16 at the Darány study area.

#### 2.2.3. Badger Habitat Composition Estimation

To estimate the habitat composition in the surrounding (400 m radius buffer) of inhabited burrows, we used Quantum GIS 3.31.1 software and Corine Land Cover 100 (version: 2018) layer [30]. When creating the database, we first intersected the CLC100 overlay with the buffer areas. As a result, we obtained the overlay of the buffers based on CLC categories [31]. In the next step, random points were placed (repeated five times) on the study areas. The number of random points were equal to the number of inhabited burrows found in the given study. In case of random points, the habitat composition was estimated using the buffer (400 m radius) method. The sample size (number of inhabited burrows) was enough only in the case of badgers for this estimation.

### 2.3. Data Analysis

#### 2.3.1. Analysing the Wildlife Camera Results

All camera recordings were analysed and organized into separate folders according to burrows. The analysis of the images enabled us to identify the number of adult individuals occupying each burrow from which we were able to determine the size of the annual population. Furthermore, we also observed the number of cubs and we were able to calculate the average number of cubs in the burrows, as well as the number of cubs in all inhabited burrows. The minimum number of individuals living in the area can be calculated from the sum of the number of adult individuals and cubs.

#### 2.3.2. Analysing the Badger Habitat Composition Results

For statistical analysis, the inhabited burrows were divided into two groups, which are the burrows with cubs and the burrows without cubs. Using the Mann–Whitney U-test, the size of each CLC category found in the surrounding of burrows with cubs was compared to the categories found in the surrounding of burrows without cubs and random points. For statistical analysis, we used Microsoft Excel and the GraphPad Instat 3 statistical program.

## 3. Results

### 3.1. Burrow Density

#### 3.1.1. Drávaszentes

We surveyed 23 burrows in the study area in Drávaszentes, 15 of which belonged to badgers and 7 to foxes (Figure 1). Of the 15 badger burrows, 11 were inhabited, while of the 7 fox burrows, 4 were inhabited. Out of these, 2 burrows were occupied by both species. Based on these data, it can be calculated that the number of inhabited burrows was 1.05 burrows/100 hectares for badgers and 0.38 burrows/100 hectares for foxes. The minimum distance of badger burrows from each other was 627 m and the maximum distance was 5407 m, while the average distance was 2524 m. The minimum distance between two fox burrows was 1346 m and the maximum distance was 4173 v meters, while the average distance was 2722 m. The distance between the nearest fox burrow and badger burrow was 303 m.

#### 3.1.2. Darány

In the study area in Darány, we surveyed 42 burrows, of which 24 belonged to badgers and 18 belonged to Cannidae species (Figure 2). Of the 24 badger burrows, 14 were inhabited and 0 of the 16 fox burrows were inhabited, while both of the 2 golden jackal burrows were inhabited. From these figures, it can be calculated that the number of inhabited burrows per 100 hectares is 1.43 for badger, 0 for fox, and 0.2 for golden jackal. The minimum distance of badger burrows from each other was 186 m and the maximum distance was 3757 m, while the average distance was 1722 m. For Jackal, the two burrows that we found were 2231 m away. The distance between the nearest jackal burrow and badger burrow was 407 m.

### 3.2. The Estimated Number of Individuals per Burrow by Wildlife Cameras

#### 3.2.1. Drávaszentes

Based on wildlife camera recordings, the following minimum numbers of individuals were observed in the 11 badger setts examined. Cubs were observed in eight cases (one cub in seven cases, two cubs in one case). Based on these data, it was possible to calculate the average number of cubs per burrow, which was 1.13 cubs/burrow, resulting in 0.82 cubs/burrow when projected onto all inhabited burrows. The data obtained show that no litters larger than two cubs were observed during the study. In total, we counted nine cubs. The number of adult individuals observed in inhabited burrows was 15, which means that there were 1.36 adult individuals/burrow. Adding the number of adult individuals and cubs together gives a value of 2.18 individuals/burrow. Thus, our estimated minimum population is 24 individuals, of which 15 are the core population.

Based on data provided by wildlife cameras, the following minimum numbers of individuals were observed in the four fox burrows studied. We observed fox cubs in three cases using wildlife cameras (one cub in one case, three cubs in two cases). Thus, it was possible to calculate that the average number of cubs per burrow with cubs was 2.33 cubs/burrow, while the number of cubs per burrow was 1.75 cubs/burrow for all occupied burrows. The number of adults observed in the burrows was 1.25 adults/burrow. We recorded a total of seven cubs. Adding the number of adults (5) and cubs together gives a result of three individuals per burrow. Thus, our estimated minimum population is 13 individuals and our core population is 5 individuals.

#### 3.2.2. Darány

Based on wildlife camera recordings, the following minimum numbers of individuals were observed in the 14 badger setts studied. In this area, we observed cubs on five occasions (one cub in two cases, two cubs in three cases). From the data, it was calculated that the average number of cubs per burrow was 1.6 cubs/burrow, which, broken down for all inhabited burrows, gave a value of 0.58 cubs/burrow. No litters larger than two cubs were observed in this study area. We recorded a total of eight cubs. In terms of adult individuals, the value per burrow was 1.57 individuals. After adding up the number of adult individuals and cubs, we obtained a value of 2.15 individuals/burrow. Thus, our estimated minimum population is 30 individuals, of which 22 are the core population.

Based on wildlife camera recordings, the following minimum numbers of individuals were observed at the two jackal burrows studied. In the case of golden jackals, only adults were observed in the area (2 cases). Thus, the number of adult individuals observed is 1 adults/burrow. Therefore, our minimum estimated number of individuals is two, of which two are part of our core population.

### 3.3. Habitat Composition Results for Badger Burrows

#### 3.3.1. Drávaszentes

At the Drávaszentes study site, a statistically significant difference was found in one case (out of six) among the five examined habitat types between burrows with cubs and the “random 5” points. The difference can be seen in the case of non-irrigated arable land (CLC 211) (Table A1). Descriptive statistics also show that the median value for this habitat type in the surrounding of the burrows with cubs (18.42 hectare) was much higher than that measured for “random 5” points (0.64 hectare) (Table A3). This might indicate that in this area, badger breeding burrows are more freqently located in enviroments where proportions of CLC 211 are high within the immediate 400 m vicinity. In other cases (Broad-leaved forest—CLC 311, Discontinious urban fabric—CLC 112, Pastures—CLC 231, Land occupied by agriculture with significant natural vegetation—CLC 243), no significant difference was detectable (*p* > 0.05), or the test could not be performed due to lack of data.

#### 3.3.2. Darány

In the Darány area, a significant pattern was obseved regarding forest habitats among the six examined habitat types. Mixed forest (CLC 313) habitats were found in significantly higher proportions in the environment of burrows with cubs compared to random points in multiple cases. Compared to “random 1”, the difference was significant (Table A2) where the median value for burrows with cubs (23.61 hectare) exceeded the random points (9.53 hectare). Similary, a significant difference was found for the “random 2” group, again favoring burrows with cubs (median: 23.61 to 8.49 hectare) (Table A4). These result suggest that in Darány, mixed forest (CLC 313) might be considered an important habitat for burrows with cubs. For other habitat types (Non-irrigated arable land—CLC 211, Broad-leaved forest—CLC 311, Coniferous—CLC 312, Mixed forest—CLC 313, Transitional woodland-shrub CLC 324, Water bodies—CLC 512), the examined comparison did not show statistically verifiable differences.

## 4. Discussion

### 4.1. Burrow

Our study shows that the total burrow count was a good method to analyse European badger distribution, but it is insufficient for accurate golden jackal and red fox monitoring because they do not always use burrows for raising cubs and the foxes can use more burrows simultaneously to rear cubs [15,16]. The recorded badger burrow densities (1.05–1.43 inhabited burrows/100 hectares) align with the species behaviour of using burrows for social interactions between different members of a badger group, breeding and sheltering over winter [25,32,33]. This supports the observation that foxes and jackals have high plasticity in using burrows and can utilize surface covers or structures (abandoned cellars) that can evade standard burrow surveys.

If we compare our results of badger burrow density, we can see that our recorded densities of 1.05 burrows/100 ha in Drávaszentes and 1.43 burrows/100 hectares in Darány exceed the values of previous studies for the Darány study area [34] and exceed the values of a study from the Drávaszentes study area [35]. Our result is higher than what was estimated at East Hungary [36].

For red foxes, our observed burrow density of 0.38 burrows in Drávaszentes compared to previous studies at the same location gave a similar result [35]. At Darány study area, we recorded 0 burrows, but a previous study reported an amount of 0.4 burrow/100 hectares [34]. If compare the results we obtained with that from South-Eastern Hungary, we obtain values between 0.23 and 0.47 which on average is roughly the same as our results [15].

For the golden jackal, if we compare the results we obtained in the Darány study area with the result obtained at the same area (0.3 burrow/100 hectare), we achieve a similar result. In East Hungary, two other studies have been performed on jackals (0.32–0.34 family/100 hectares) with a higher result [15].

### 4.2. Minimum Population and Litter Size Recorded by Wildlife Cameras

We need to clarify that our results of litter sizes only show the cubs that left the burrows and that the cameras have the following flaws: the camera cannot see the whole area of the burrow, only the entrance; it can only detect movement up to a certain distance and does not always turn on for movement; sometimes when it detects movement, the whole animal is not on the picture.

The badger litter sizes we obtained (1.13–1.6) can be compared with the results of a previous study in the Drávaszentes study area (1.29 cub/burrow) which is an average of our two study areas [37]. For the red fox, our result (2.33) can be compared with a study carried out at the Drávaszentes study area (4 cub/burrow), which is a much higher number.

For jackal, we did not record any cubs but this may be because jackals can move their cubs to another burrow if they feel disturbance [34].

### 4.3. Habitat Composition at Badger

The habitat analysis showed attraction toward specific habitat features, although these varied by study sites. In Drávaszentes, badger breeding setts were associated with non-irrigated arable land (CLC 211) (*p* = 0.007 compared to random points). This likely references the “edge effect” where badgers utilize the area between cover that gives security and arable foraging grounds where they can feed.

Conversely, in Darány, we observed an attraction towards mixed forests (CLC 313) (*p* = 0.032 and *p* = 0.016 against random points). This aligns with other studies where the badger finds soil stability in these areas.

The divergence between the two study sites suggest that the badger shows behavioural plasticity and can select the most suitable habitats.

### 4.4. Jackal Effect

The fact we only found red fox burrows and litters at one of our locations and none at the other where jackals were identified suggests that the larger golden jackal population can suppress the smaller red fox population. On the other hand, badger populations are resilient to the jackal.

## 5. Conclusions

Based on the results of our study, the first hypothesis was disproved by the data. The highest number of burrows was observed for badgers. According to this result, badgers have the highest population size in the study areas. Although in Hungary, the estimated population size of red fox is two times higher than badger [4], locally, the fox population could be lower. It is possible that badgers may have a competitive advantage (larger body size and social behavior) [38]. The second hypothesis was rejected. The observed badger litter size was between 1 and 2 cubs, which is in line with the previously published data [7]. In contrast, the observed red fox litter size was between 1 and 3 cubs. These numbers are smaller than the results of previous studies [38]. The small or decreasing litter size could be an indicator of poor habitat quality, especially the lack of food sources [39]. In the case of golden jackal, the sample size (inhabited burrows) was too low (n = 2) to draw any conclusions. The third hypothesis related to habitat composition was disproved by the data as well. Comparing the area size of the given habitat types between burrows with cubs and random points, we found few differences. In the rest of the cases, the habitat composition was the same. Further studies should focus on small-scale [39] microhabitat composition of burrows using greater sample sizes and detailed analysis.

## Data Availability

The data presented in this study are available on request from the corresponding author.

## Appendix A

**Table A1:** The results of Mann–Whitney U-test in the case of Drávaszentes study area. Legend: 112-Discontinuous urban fabric, 211-Non-irrigated arable land, 231-Pastures, 243-Land principally occupied by agriculture, with significant areas of natural vegetation and 311-Broad-leaved forest.

Habitat Type	Comparison	U Stat	U’ Stat	*p* Value	n
112	Burrows with cubs—Burrows with no cubs	7.000	17.000	0.355	11
	Burrows with cubs—random 1	31.000	33.000	0.957	16
	Burrows with cubs—random 2	32.000	32.000	0.957	16
	Burrows with cubs—random 3	24.000	40.000	0.423	16
	Burrows with cubs—random 4	28.000	36.000	0.700	16
	Burrows with cubs—random 5	31.000	33.000	0.957	16
211	Burrows with cubs—Burrows with no cubs	11.000	13.000	0.921	11
	Burrows with cubs—random 1	23.000	41.000	0.382	16
	Burrows with cubs—random 2	21.000	43.000	0.279	16
	Burrows with cubs—random 3	21.000	43.000	0.279	16
	Burrows with cubs—random 4	24.000	40.000	0.442	16
	Burrows with cubs—random 5	7.000	57.000	0.007	16
231	Burrows with cubs—Burrows with no cubs	6.000	18.000	0.279	11
	Burrows with cubs—random 1	23.500	40.500	0.400	16
	Burrows with cubs—random 2	26.500	37.500	0.598	16
	Burrows with cubs—random 3	29.500	34.500	0.833	16
	Burrows with cubs—random 4	29.500	34.500	0.833	16
	Burrows with cubs—random 5	23.000	41.000	0.371	16
243	Burrows with cubs—Burrows with no cubs	0.000	0.000	0.000	11
	Burrows with cubs—random 1	29.000	35.000	0.783	16
	Burrows with cubs—random 2	0.000	0.000	0.000	16
	Burrows with cubs—random 3	0.000	0.000	0.000	16
	Burrows with cubs—random 4	0.000	0.000	0.000	16
	Burrows with cubs—random 5	29.000	35.000	0.783	16
311	Burrows with cubs—Burrows with no cubs	6.000	18.000	0.279	11
	Burrows with cubs—random 1	32.000	32.000	1.000	16
	Burrows with cubs—random 2	29.000	35.000	0.798	16
	Burrows with cubs—random 3	30.000	34.000	0.879	16
	Burrows with cubs—random 4	32.000	32.000	1.000	16
	Burrows with cubs—random 5	30.000	34.000	0.879	16

**Table A2:** The results of Mann–Whitney U-test in case of Darány study area. Legend: 211-Non-irrigated arable land, 231-Pastures, 311-Broad-leaved forest, 312-Coniferous forest, 313-Mixed forest, 324-Transitional woodland-shrub and 512-Water bodies.

Habitat Type	Comparison	U Stat	U’ Stat	*p* Value	n
211	Burrows with cubs—Burrows with no cubs	24.500	25.500	1.000	15
	Burrows with cubs—random 1	4.500	20.500	0.116	10
	Burrows with cubs—random 2	11.000	14.000	0.828	10
	Burrows with cubs—random 3	11.000	14.000	0.828	10
	Burrows with cubs—random 4	11.000	14.000	0.828	10
	Burrows with cubs—random 5	11.000	14.000	0.828	10
231	Burrows with cubs—Burrows with no cubs	0.000	0.000	0.000	15
	Burrows with cubs—random 1	0.000	0.000	0.000	10
	Burrows with cubs—random 2	0.000	0.000	0.000	10
	Burrows with cubs—random 3	0.000	0.000	0.000	10
	Burrows with cubs—random 4	0.000	0.000	0.000	10
	Burrows with cubs—random 5	0.000	0.000	0.000	10
311	Burrows with cubs—Burrows with no cubs	18.000	32.000	0.440	15
	Burrows with cubs—random 1	5.000	20.000	0.151	10
	Burrows with cubs—random 2	3.000	22.000	0.056	10
	Burrows with cubs—random 3	12.000	13.000	1.000	10
	Burrows with cubs—random 4	12.000	13.000	1.000	10
	Burrows with cubs—random 5	9.000	16.000	0.548	10
312	Burrows with cubs—Burrows with no cubs	0.000	0.000	0.000	15
	Burrows with cubs—random 1	0.000	0.000	0.000	10
	Burrows with cubs—random 2	0.000	0.000	0.000	10
	Burrows with cubs—random 3	0.000	0.000	0.000	10
	Burrows with cubs—random 4	0.000	0.000	0.000	10
	Burrows with cubs—random 5	0.000	0.000	0.000	10
313	Burrows with cubs—Burrows with no cubs	25.000	25.000	1.000	15
	Burrows with cubs—random 1	2.000	23.000	0.032	10
	Burrows with cubs—random 2	1.000	24.000	0.016	10
	Burrows with cubs—random 3	11.000	14.000	0.841	10
	Burrows with cubs—random 4	8.000	17.000	0.421	10
	Burrows with cubs—random 5	8.000	17.000	0.421	10
324	Burrows with cubs—Burrows with no cubs	0.000	0.000	0.000	15
	Burrows with cubs—random 1	0.000	0.000	0.000	10
	Burrows with cubs—random 2	0.000	0.000	0.000	10
	Burrows with cubs—random 3	0.000	0.000	0.000	10
	Burrows with cubs—random 4	0.000	0.000	0.000	10
	Burrows with cubs—random 5	0.000	0.000	0.000	10
512	Burrows with cubs—Burrows with no cubs	9.000	16.000	0.521	15
	Burrows with cubs—random 1	9.000	16.000	0.521	10
	Burrows with cubs—random 2	7.000	18.000	0.284	10
	Burrows with cubs—random 3	6.000	19.000	0.199	10
	Burrows with cubs—random 4	9.000	16.000	0.521	10
	Burrows with cubs—random 5	10.000	15.000	0.674	10

**Table A3:** The results of descriptive statistics in hectares in the case of Drávaszentes study area. Legend: 112-Discontinuous urban fabric, 211-Non-irrigated arable land, 231-Pastures, 243-Land principally occupied by agriculture, with significant areas of natural vegetation and 311-Broad-leaved forest.

Category	Habitat Type	MIN	Q1	MED	Q3	MAX
Burrows with cubs	112	0.00	0.00	0.00	1.56	22.11
	211	5.55	8.87	18.42	21.44	37.77
	231	0.00	0.00	4.62	12.94	23.82
	243	0.00	0.00	0.00	0.27	3.76
	311	7.58	11.36	18.43	26.00	42.06
Burrows with no cubs	112	0.00	3.16	6.32	6.65	6.98
	211	14.78	16.91	19.03	19.56	20.09
	231	13.79	16.19	18.58	18.97	19.35
	243	0.00	0.00	0.00	0.00	0.00
	311	3.79	7.44	11.09	13.98	16.87
random 1	112	0.00	0.00	0.00	0.00	2.57
	211	0.00	0.00	8.70	8.97	11.64
	231	0.00	0.00	4.07	15.66	20.12
	243	0.00	0.00	0.00	0.00	0.00
	311	4.09	4.09	14.95	17.25	21.51
random2	112	0.00	0.00	0.00	2.05	11.92
	211	0.00	2.54	9.19	21.96	30.28
	231	0.00	0.00	10.36	18.01	36.74
	243	0.00	0.00	0.00	0.00	0.00
	311	1.39	16.87	21.00	31.56	45.53
random 3	112	0.00	0.00	3.84	8.32	13.51
	211	0.00	0.32	9.72	17.91	43.72
	231	0.00	0.00	4.99	14.11	18.51
	243	0.00	0.00	0.00	0.00	0.00
	311	0.00	10.18	19.80	29.25	50.22
random 4	112	0.00	0.00	0.00	0.00	14.78
	211	0.00	0.90	12.92	20.12	48.92
	231	0.00	0.00	8.20	18.62	28.89
	243	0.00	0.00	0.00	0.00	0.00
	311	1.30	7.86	20.35	39.85	49.04
random 5	112	0.00	0.00	0.00	0.92	17.63
	211	0.00	0.00	0.64	3.12	27.23
	231	0.00	0.86	15.39	36.75	46.52
	243	0.00	0.00	0.00	0.00	4.89
	311	0.04	8.39	16.14	44.99	49.47

**Table A4:** The results of descriptive statistics in hectares in the case of Darány study area. Legend: 211-Non-irrigated arable land, 231-Pastures, 311-Broad-leaved forest, 312-Coniferous forest, 313-Mixed forest, 324-Transitional woodland-shrub and 512-Water bodies.

Category	Habitat Type	MIN	Q1	MED	Q3	MAX
Burrows with cubs	211	0.00	0.00	0.00	0.09	8.12
	311	9.56	10.02	19.58	25.38	27.70
	312	0.00	0.00	0.00	0.00	0.00
	313	14.39	18.88	23.61	32.45	40.66
	512	0.00	0.00	5.97	7.04	7.66
Burrows with no cubs	211	0.00	0.00	0.00	4.78	23.10
	311	1.54	6.75	15.03	23.40	30.15
	312	0.00	0.00	0.00	0.00	25.37
	313	0.00	16.35	24.33	36.74	48.46
	512	0.00	0.00	0.11	1.69	6.21
random 1	211	0.00	0.49	17.34	20.10	23.80
	231	0.00	0.00	0.00	0.00	0.23
	311	16.65	24.45	28.45	28.77	30.11
	312	0.00	0.00	0.00	0.00	0.00
	313	0.00	8.43	9.53	11.95	20.96
	512	0.00	0.00	0.00	0.00	9.82
random2	211	0.00	0.00	0.00	0.00	32.57
	231	0.00	0.00	0.00	0.00	7.30
	311	10.36	29.10	31.24	34.94	41.73
	312	0.00	0.00	0.00	3.51	12.19
	313	0.00	0.00	8.49	9.17	15.84
	324	0.00	0.00	0.00	1.77	6.12
	512	0.00	0.00	0.00	0.00	6.80
random 3	211	0.00	0.00	0.00	0.00	29.80
	231	0.00	0.00	0.00	0.00	1.66
	311	7.38	10.38	14.09	27.33	43.76
	312	0.00	0.00	0.00	0.00	0.11
	313	4.67	6.35	22.89	39.11	42.83
	324	0.00	0.00	0.00	0.00	0.00
	512	0.00	0.00	0.00	0.00	0.73
random 4	211	0.00	0.00	0.00	0.00	11.23
	231	0.00	0.00	0.00	0.00	0.00
	311	1.35	11.60	21.62	24.53	35.37
	312	0.00	0.00	0.00	0.00	36.81
	313	12.04	14.46	14.84	27.29	27.29
	324	0.00	0.00	0.00	0.00	0.01
	512	0.00	0.00	0.00	0.00	11.33
random 5	211	0.00	0.00	0.00	0.00	12.38
	231	0.00	0.00	0.00	0.00	0.00
	311	11.46	16.60	23.95	25.43	43.31
	312	0.00	0.00	0.00	0.00	0.46
	313	0.00	13.89	14.95	26.98	33.62
	324	0.00	0.00	0.00	0.00	0.00
	512	0.00	0.00	6.91	9.38	11.79
